# A Full-Spectrum Generative Lead Discovery (FSGLD) Pipeline via DRUG-GAN: A Multiscale Method for Drug-like/Target-specific Compound Library Generation

**DOI:** 10.21203/rs.3.rs-6516504/v1

**Published:** 2025-05-12

**Authors:** Beihong Ji, Matthew Brock, Yuhui Wu, Yuemin Bian, Xibing He, Junmei Wang

**Affiliations:** 1 Hangzhou Institute of Medicine, Chinese Academy of Sciences, Hangzhou, 310018, Zhejiang, China.; 2 Department of Pharmaceutical Sciences and Computational Chemical Genomics Screening Center, School of Pharmacy, University of Pittsburgh, Pittsburgh, PA 15261, USA.; 3 Center for the Development of Therapeutics, Broad Institute of MIT and Harvard, Cambridge, MA 02142, United States

## Abstract

We present the Full-Spectrum Generative Lead Discovery (FSGLD), a deep learning-driven pipeline for efficient drug lead identification. FSGLD integrates generative modeling with molecular docking, molecular dynamics simulations, ligand-residue interaction profile, MM-PBSA, thermodynamic integration (TI), and experimental validation to bridge theoretical design and practical application. The core multiscale DRUG-GAN models enable *de novo* design for both drug-like and target-specific compounds across three scenarios: I. generation of random drug-like compounds, II. generation of target-specific compounds, III. generation of target-biased compound series featuring shared chemical structures. FSGLD significantly outperformed traditional computer-aided drug design methods in generating novel chemicals which specifically target the CB2 receptor. Additionally, a computational protocol for TI calculations was established to reduce computation time by 80–90% while maintaining accuracy. By integrating generative models with *in silico* and *in vitro* evaluation techniques, FSGLD reduces the cost of identifying novel yet viable lead compounds, offering remarkable benefits to both academic and industry.

## 1. Main

Traditional drug discovery is a challenging process since it aims to identify target-specific molecules with specific desirable properties from a large chemical space. It cost more than $2.6 billion and takes over 12 years to develop a new drug from initial idea to market approval^1,2^. In this process, a daunting challenge is to estimate the synthetical ability of 10^23^-10^60^ drug-like molecules from a large and discrete chemical space^3,4^. For the acceleration of drug discovery and development, traditional computational approaches such as virtual screening (VS) were developed^5^. However, even those *in silico* methods can speed up the drug design process, they are still limited by searching for a tiny portion of chemical space of a given compound library^6,7^. Therefore, identification of novel druglike chemicals from the huge chemical space is urgently needed.

Artificial intelligence has come to the view of researchers and gradually being integrated into all kinds of computer-aided drug design approaches, especially in our effort of expanding druglike chemical space. At the early stage, Simplified Molecular Input Line Entry System (SMILES) string, a textual descriptor of a molecule was applied to develop deep generative models for drug design. Until today, SMILES is still the commonly adopted representation of a molecule for generative models^8–11^. However, SMILES was not designed for capturing the structural similarity between molecules. For example, two different SMILES strings might represent molecules with the same chemical structures^12,13^. Alternatively, molecular graph for which molecular validity can be easily assessed, can also be applied to describe molecular structures in generative modeling^13–16^. Nevertheless, generative models based on molecular graphs still face critical limitations, including training complexity particularly for large molecule and only generating molecules with the same numbers of atoms of input ones^12^. More recently, 3D molecular representations emerge, where the atomic feature space is represented by atoms with spatial coordinates alongside charges and atom types^17^. However, these methods can be computationally demanding and may not necessarily enhance the quality of generative outcomes.

In this study, we decided to use simple and plain molecular fingerprint (FP) as the molecular descriptor to build the generative models. While the molecular FP representation has an intrinsic limitation, i.e., it is difficult and unambiguously translate the generated FPs into molecular structures, it entails some advantages over the other representations as well. This simple representation is more suitable to quickly generate large amounts of unique FPs. When FP-based generative modeling is in combination with fast similarity search, one can narrow down the search space and quickly identify the most promising candidates. This combination not only enhances the efficiency of molecular generation but also addresses the synthetic feasibility concerns as long as the molecular databases collecting the existing molecules. Some works devoted to developing deep learning models using FP as input features in drug development and discovery are listed as follows. Kadurin et al. adopted molecular FP as input features to develop a deep adversarial model for identification and generation of new compounds utilizing available chemical and biological data^18^. Yuemin et al. used four kinds of molecular FPs, Molecular ACCess System (MACCS), Extended-Connectivity Fingerprint 6 (ECFP6), AtomPair and AtomPair Count to build a Generative Adversarial Network (GAN) to screen out and develop novel compounds for cannabinoid receptors^19^.

Many deep generative algorithms have been introduced and successfully applied in *de novo* drug design, such as recurrent neural networks (RNNs), autoencoder (AE)-based networks, generative adversarial networks (GANs), and graph neural networks (GNNs). Recently, flow-based and diffusion-based generative models have also emerged ^20,21^, leveraging E(n)-Equivariant GNNs to achieve SE(3)-equivariance in molecular generation^22^. Among these methods, GANs still offer distinct advantages that make them particularly suitable for molecular design. The concept of GANs was initially proposed by Goodfellow et al. in 2014^23^, which introduces a competitive framework for two neural network models, the generator and discriminator, being engaged in a dynamic adversarial process. GANs have been successfully applied in a flurry of fields, mainly including the generation of images, audios and videos^24,25^. The first implementation of GANs in molecule *de novo* design was achieved by Gabriel el al. in 2018^10^. The researchers extended SeqGan with reinforcement learning to develop an objective-reinforced generative adversarial network (ORGAN) for the design of molecules. In 2018, Nicola and Thomas introduced MolGAN model for the generation of small-sized molecules using molecular graphs ^14^. Oleksii et al. proposed an autoencoder combined with a GAN model for *de novo* molecular design in 2019^26^. In 2020, Łukasz et al. developed a CycleGAN-based model that can generate optimized compounds which are structurally similar to the original ones^27^. And in the same year, Oscar et al. reported an approach by utilizing conditional GANs to generate novel molecules based on particular gene expression signatures^28^. In 2022, Maryam et al. proposed a framework based on Feedback GAN that includes optimization strategy by incorporating Encoder–Decoder, GAN, and Predictor deep models interconnected with a feedback loop^29^.

In this study, we built a series of (conditional) deep convolutional GAN ((c)dcGAN) models for *de novo* molecular design. Our main purpose is to discover novel chemicals served as drug leads which are not only drug-like but also target-specific. Moreover, the compound-series model can also be applied in drug lead optimization via exploring R-groups. Those models were collected in our DRUG-GAN platform. We applied this platform in three scenarios: I. to generate novel drug-like compounds; II. to generate target-biased novel compounds; III. to generate target-biased compounds which share common chemical structures. For all of three scenarios, the deep convolutional neural networks (DCNNs)^30–33^ were applied to construct both the discriminator and generator of a GAN model. It is noted that for the generation of target-specific compounds, a conditional dcGAN (cdcGAN) model was utilized ^34^. Conditional GAN, first proposed by Mehdi and Simon in 2014^35^, is an extension of the original GAN concept. This model introduces conditional input to guide the generation of data belonging to a specific class. In our work, we have innovatively integrated deep convolutional networks with the framework of conditional GAN, to develop conditional dcGAN (cdcGAN) models for Scenarios II and III. This advanced model can better guide the generation of target-biased active compounds by including both active and inactive CB2 ligands in the training data. Although cdcGAN was previously put forth by several studies^36–39^, our work represents the first application of applying this model type in generative chemistry. The cdcGAN model was utilized for the *de novo* design of both CB2 compounds and compound series. The designed novel FPs were applied as queries to obtain top hits in ChEMBL and ZINC databases, which were further evaluated by a series of hierarchical molecular modeling workflow consisting of multiscale molecular modeling techniques: molecular docking, ligand-residue interaction profiles (LRIP) using previously developed machine learning-based scoring function ^40^, molecular dynamics (MD) simulation, molecular mechanics-Poisson Boltzmann surface area (MM-PBSA) free energy calculations^41–46^, and thermodynamic integration (TI) alchemical free energy calculations ^47–49^.

**Fig. 1:**
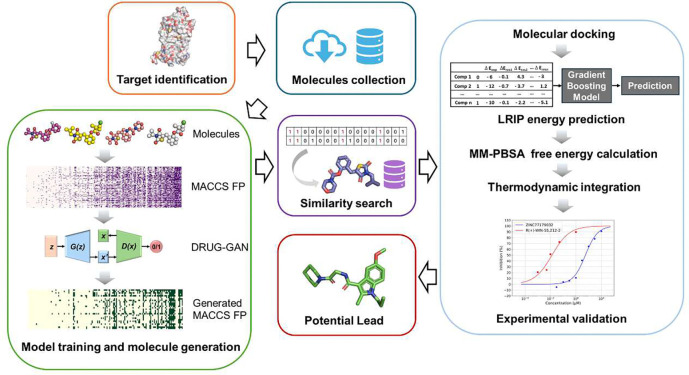
An overview of Full-Spectrum Generative Lead Discovery (FSGLD) pipeline.

To the best of our knowledge, Full-Spectrum Generative Lead Discovery (FSGLD) is the first comprehensive pipeline not only leveraging generative models to design drug-like and target-biased compounds but also systematically screening and validating those novel compounds via a hierarchical of *in silico* processes and *in vitro* experimental validation (Fig. 1). Our pipeline establishes an end-to-end procedure that bridges the gap between theoretical design and practical application, making it highly efficient and actionable. By combining state-of-the-art generative modeling with robust molecular modeling techniques, we provide a practical and scalable framework that dramatically reduces both the time and cost in preclinical drug discovery. Importantly, the compounds generated by this pipeline are not just theoretical candidates but are viable for experimental validation, offering tangible benefits for both researchers and industry professionals.

## 2. Results

### 2.1 Prediction results for discriminators

**Fig. 2:**
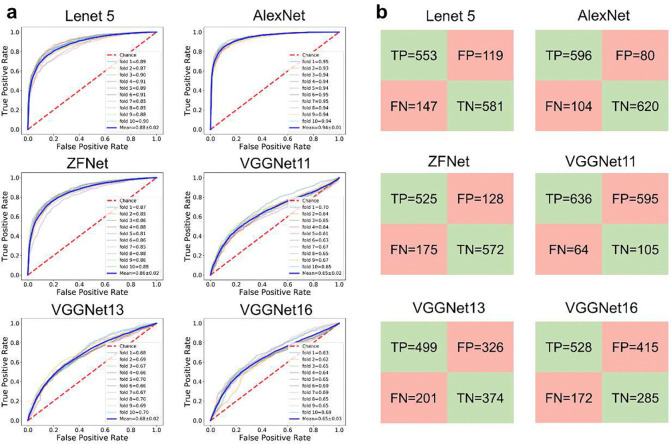
Performance of discriminator models. (a) ROC curves of ten-fold cross validation; (b) Confusion matrixes list the average values of true positives (TP), true negative (TF), false positive (FP) and false negative (FN).

The dataset for the initial training of the discriminator in dcGAN model is composed of 7002 active and 9998 inactive molecules. While the 7002 active molecules are drugs collected by DrugBank^50^, 9998 inactive molecules were randomly selected from ZINC database^51^ with a restriction that the distributions of molecular weight (MW) and the logarithm of the partition coefficient between water and octanol (logP) of the active and inactive datasets are essentially similar. We trained the discriminator using different DCNN architectures and the model performance was summarized in Fig. 2 and **Table S1**. In total six DCNN architectures, namely LeNet 5, AlexNet, ZFNet, VGGNet11, VGGNet13, VGGNet16, were investigated with the aim to identify the DCNN architecture which can best fit the input features to achieve the best classification accuracy. Fig. 2a shows the receiver operating characteristic (ROC) curves on test sets for the six DCNN models. The model performance of a classifier can be measured by the area under the ROC curve (AUC). The AlexNet models achieved the best average AUC value of 0.94, outperforming the DCNN models based on the other architectures (**Table S1**). AlexNet model was first introduced by Hinton and his co-workers in the ImageNet competition in 2012^31^. Their AlexNet-based DNN model showed excellent performance and took the first place in the competition. According to the confusion matrixes shown in Fig. 2b, DCNN models trained using the Lenet 5, AlexNet and ZFNet architectures have better performance than the three VGGNet-based architectures since the former three have much more true positives (TP) (>550) and true negatives (TN) (>550), and fewer false positives (FP) and false negatives (FN). These results suggested that the number of convolutional layers in a DCNN model is not positively correlated with the model performance.

Besides ROC-AUC, **Table S1** also summarized the average values of other key performance metrics, including accuracy, F1-score, precision and recall scores, for all DCNN models on the test set. According to this table, AlexNet-based models were ranked No 1 on all performance metrics except for recall, which were ranked the second place; LeNet 5-based models performed slightly worse with the second-highest AUC, accuracy, F1-score and precision scores.

### 2.2 Scenario I: generation of drug-like compounds

Regarding the results evaluated for the discriminator model, as aforementioned, the DCNN models trained with the Alexnet architecture achieved the best performance in distinguishing the active FP from the inactive ones. Therefore, Alexnet was selected as the DCNN architecture to construct discriminators for all DRUG-GAN models. The architecture of a generator was essentially created by reversing the convolutional architecture of the discriminator. After training, 10,000 molecular FP were generated using the dcGAN model and their quality was compared against the 7002 drug FP in the training set.

A comparison of the generated FP by DRUG-GAN and published models (ORGAN, OR(W)GAN, and ORGANIC) was presented in Table 1. Note that we converted the generated SMILES strings by the published models into FPs for comparison. Overall, DRUG-GAN outperformed the benchmark models across several key metrics. Notably, DRUG-GAN achieved perfect uniqueness and novelty scores (1.00 for both), indicating every generated fingerprint was unique and novel when compared to the training data. The DRUG-GAN’s diversity score (0.73) was comparable to those of ORGAN’s (0.77) and ORGANIC’s (0.76), slightly higher than that of OR(W)GAN (0.63). In terms of average similarity to the 7002 drugs, DRUG-GAN outperformed the other models with a score of 0.25, reflecting the efficacy of its training process and its capacity to generate molecules similar to real drugs.

**Fig. 3:**
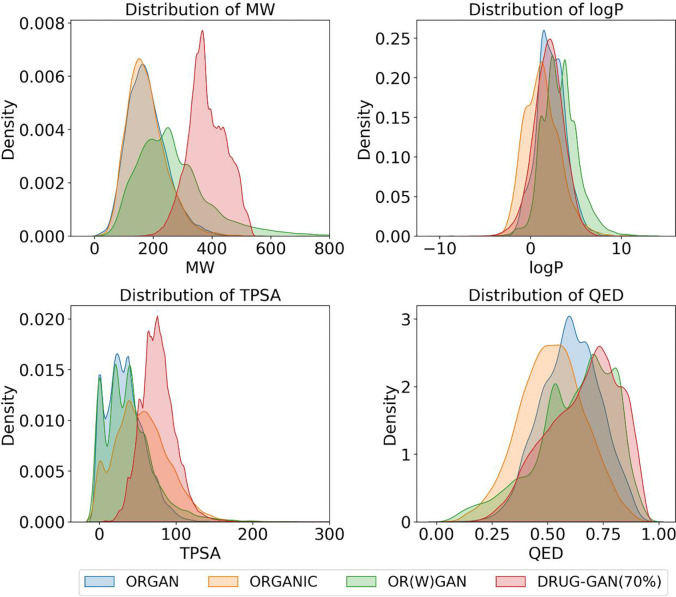
Distributions of Molecular Properties for Molecular Datasets Generated by DRUG-GAN with TS ≥ 70% and Three Published Generative Models.

One limitation using FPs in generative modeling is to convert the generated molecular fingerprints into molecular structures. To address this limitation, we adopted a similarity search to quantitatively evaluate the generated FPs in compound databases. For the 10,000 generated FPs, we have identified 493201, 30851 and 928 compounds that have at least 70%, 75% and 80% maximum structural similarity measured by Tanimoto score (TS) to compounds in the ZINC database. It is pointed out that not all the SMILES strings generated by the three published models are valid, among the 10,000 SMILES strings, 8235, 5530 and 9146 SMILES strings are valid and unique for ORGAN, OR(W)GAN and ORGANIC, respectively. Next, we calculated important molecular properties for the resulting molecules for each model. As shown in Table 2 and Fig. 3, most molecules from each model adhered to the Lipinski’s rule of five, however, the molecules resulting from DRUG-GAN demonstrated better druglike properties, especially for those obtained using the 70% similarity threshold. We did a statistic analysis on 2252 approved drugs by FDA that have molecular weights (MW) between 100 and 1000, the mean MW, logP and TPSA are 358.0, 2.8 and 89.3, respectively. Apparently, molecules resulting from DRUG-GAN exhibited similar values of the three key druglike properties, which are 382.9, 2.0 and 76.0, respectively. Thus, those molecules are slightly larger but more soluble. In contrast, molecules generated by the three published models are significantly smaller than approved drugs with MW ranged from 177 to 267.

**Fig. 4:**
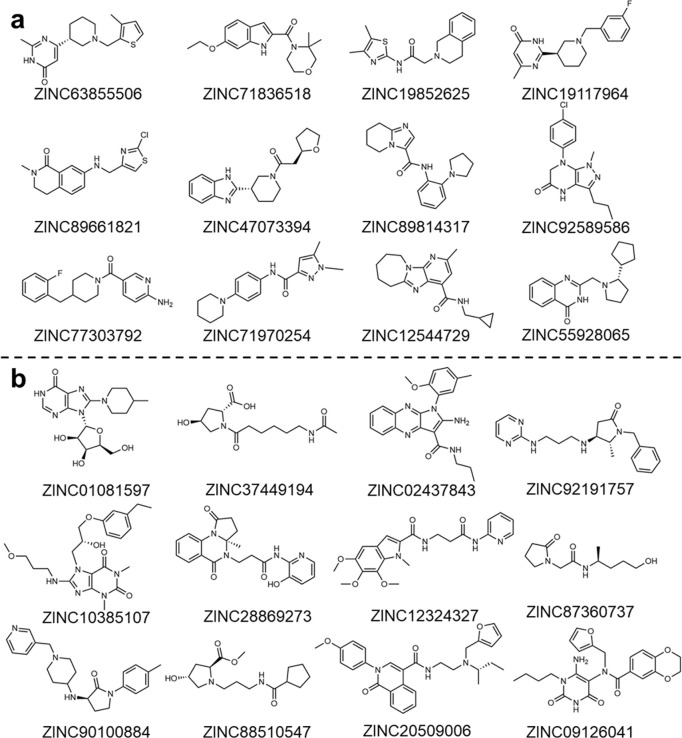
Selected Top Druglike Candidates According to QED Druglike Metrics (a) and Structural Similarity (b).

In addition, selected top candidates based on their similarities to ZINC compounds and drug-likenesses (QED) were listed in **Tables S2-S3** and Fig 4. These candidates showed a favorable balance of molecular weight, lipophilicity, and polar surface area, suggesting promising drug-like characteristics. Moreover, those compounds are structurally diverse, suggesting that DRUG-GAN is able to explore broad chemical space. Thus, our DRUG-GAN model integrating similarity search is a viable protocol to construct high-quality screening compound library for drug discovery.

### 2.3 Scenario II: target-biased compound generation

**Fig. 5:**
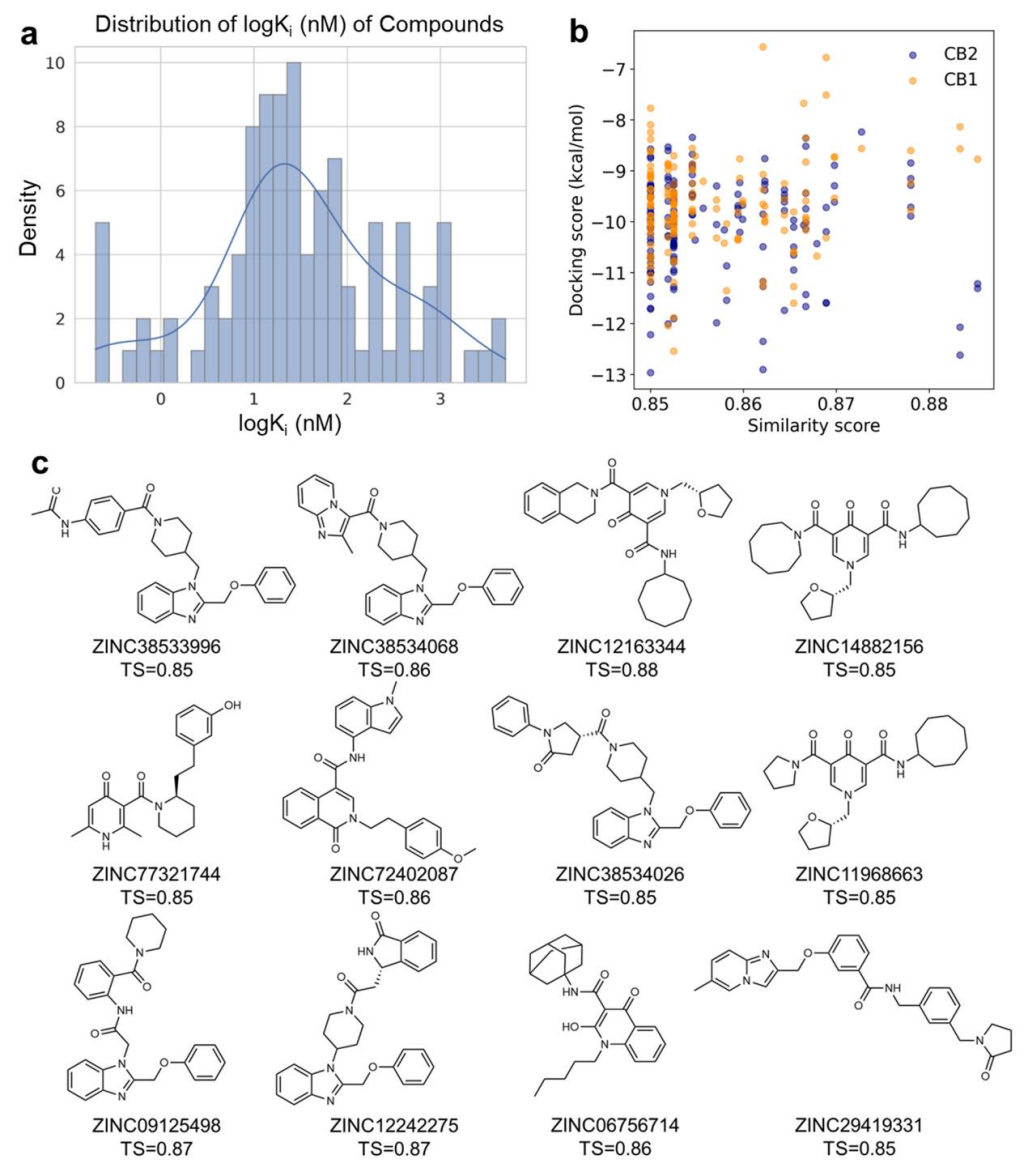
The Performance of DRUG-GAN in Senario II. **a.** The distribution of logK_i_ of searched CB2 active compounds from the ChEMBL database. **b.** The scatter plot of the docking scores versus similarity scores for searched ZINC compounds targeting to CB1 and CB2 receptors. **c.** Structures of top CB2 active candidates in ZINC database.

In addition to generating druglike candidates, DRUG-GAN also has the capability to generate target-specific compounds. In the following, we took cannabinoid receptor 2 (CB2) as an example to demonstrate this application. The active and inactive CB2 compounds in the training set were used as the real data to train the model to generate CB2 active compounds. The numbers of compound hits from ChEMBL and ZINC databases using different similarity criteria are summarized in Table 3. For those compounds retrieved from ChEMBL that are at least 85% structurally similar to the generated FPs, 169 have molecular weights between 100 and 500. Encouragingly, 104 out of 169 have been recognized as active CB2 ligands in bioassays (K_i_ < 10 μM). The mean K_i_ values of these compounds are 267.22 nM, with a range spanning from 0.199 nM to 5000 nM. The distribution of logK_i_ values is shown in Fig. 5a. The concentration of data points in the lower range of the K_i_ spectrum indicates our DRUG-GAN protocol is able to effectively generate and retrieve the CB2 active compounds from the relevant database.

Additionally, 161 molecules were retrieved from ZINC database with TS 85% or higher against the DRUG-GAN-generated FPs. These molecules were subsequently subjected to molecular docking to predict their binding affinities to both CB1 and CB2 receptors, and the results are reported in Fig. 5b. The results revealed that DRUG-GAN can generate highly active compounds targeting CB2, the average docking score, −10.10 kcal/mol is much lower than that for a decoy molecular set (−8.29 kcal/mol). The decoy molecular set is the one used in training the discriminator in Scenario I. Moreover, we found that DRUG-GAN is also capable of generating CB2-selective compounds as there are more blue dots (CB2) distributed at the bottom than orange dots (CB1) in Fig. 5b. The top ZINC candidates with both high similarity scores and predicted binding affinity to CB1/CB2 were demonstrated in **Table S4** and Fig. 5c. It is very clear that those top compounds in **Table S4** demonstrated a very clear selectivity between CB1 and CB2 receptors. The mean difference ranged from 0.67 to 4.49 with an average of 2.41 kcal/mol. All compounds have better docking scores to CB2 than to CB1.

### 2.4 Scenario III: generation of target-biased compound series

To generate molecular fingerprints (FPs) that inherently possess MCS features, we explored known CB2 ligands collected by ChEMBL and conceived a set of common structural features shared by highly active CB2 ligands (Table 4 and **Fig. S1**). Then we added a specific mask layer to the generator so that the non-zero fingerprint bits of the MCS are reserved in the generated FPs. Next the same similarity search is employed as in Scenario II to retrieve compounds in ChEMBL and ZINC databases using four similarity criteria (TS ≥ 0.70, 0.75, 0.80 and 0.85). Given the fact that MACCS encodes molecules only using a set of 168 predefined structural keys, structural details may be missing for some molecules. To address this, we performed an additional substructure search using the 1024-key FP2 fingerprint to ensure the identified compounds truly contain the MCS.

**Fig. 6:**
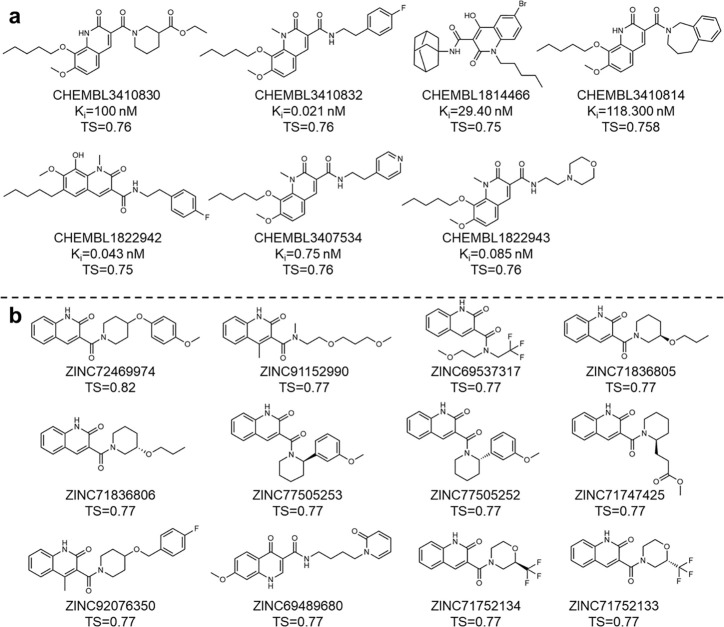
Top Compounds Containing MCS i Substructure for the CB2 receptor by searching ChEMBL database (a) and ZINC database (b).

For MCS i, 49 ChEMBL compounds were confirmed to contain this substructure and have structural similarity better than 75% against the generated fingerprint samples. Encouragingly, 7 were found to have high binding affinity for CB2 (K_i_ < 10 μM). Their exact binding affinities and structures were shown in Fig. 6a. As to ZINC database, 32 compounds identified with TS ≥ 0.75 were confirmed to possess the MCS i substructure. Top candidates along with their TS were shown in Fig. 6b. Most of these candidates demonstrated superior docking results when targeting CB2 receptor compared to CB1 receptor, highlighting their potential selectivity (**Table S5**).

**Fig. 7:**
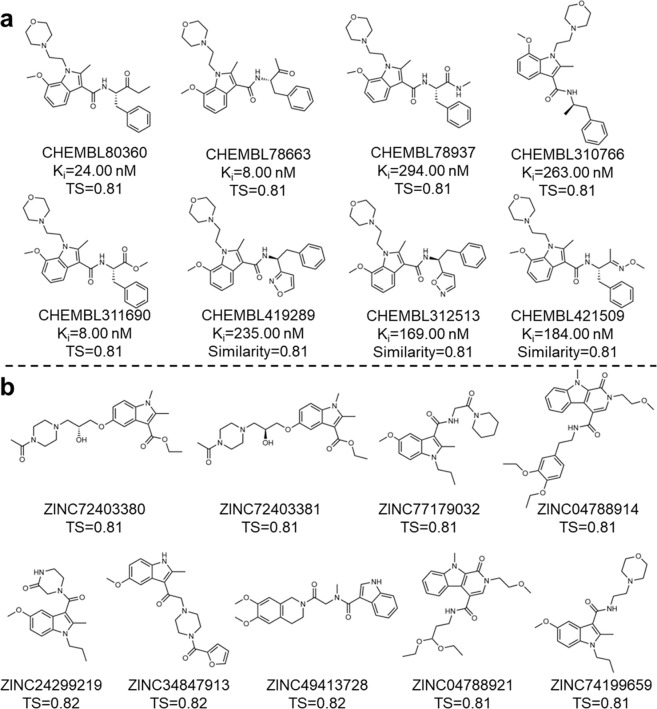
Top Compounds Containing MCS ii Substructure for the CB2 receptor by searching ChEMBL database (a) and ZINC database (b).

For MCS ii, 24 identified ChEMBL compounds exactly contain MCS ii and possess at least 80% similarity against the generated fingerprint samples. Promisingly, 8 of these compounds demonstrated strong binding affinity for CB2 target (Fig. 7a), reinforcing the potential of the generated compounds as CB2 ligands. For the ZINC database, 9 MCS ii-containing compounds have 80% or better structural similarity to the generated FPs (Fig. 7b). Similar to the MCS i feature compounds, most of these compounds demonstrated superior docking scores for the CB2 receptor than the CB1 receptor, further highlighting the ability of DRUG-GAN in generating target-specific compounds **(Table S6)**.

**Fig. 8:**
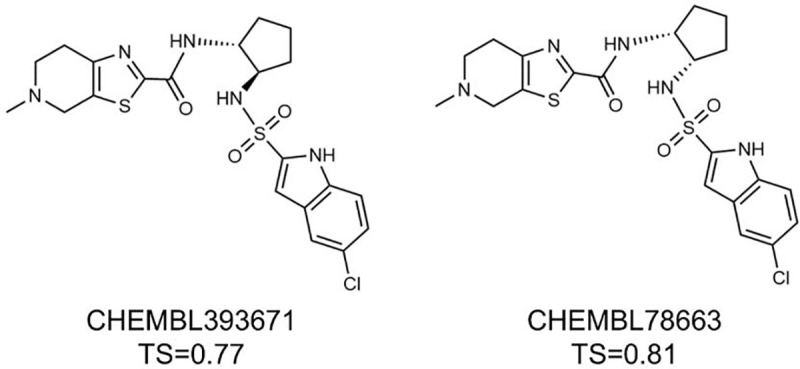
Top Compounds Containing MCS iii Substructure for the CB2 receptor by searching ChEMBL database.

As for the MCS iii feature, 165 compounds were initially retrieved from ChEMBL with a similarity score above 75%, but only 2 compounds were confirmed to have the exact MCS iii substructure using the FP2 fingerprint (Fig. 8). None of ZINC compounds with TS of 75% or higher were found to possess the exact MCS iii substructure using the FP2 fingerprint.

### 2.5 Further computational evaluation and experimental validation of the DRUG-GAN generated compounds

**Fig. 9:**
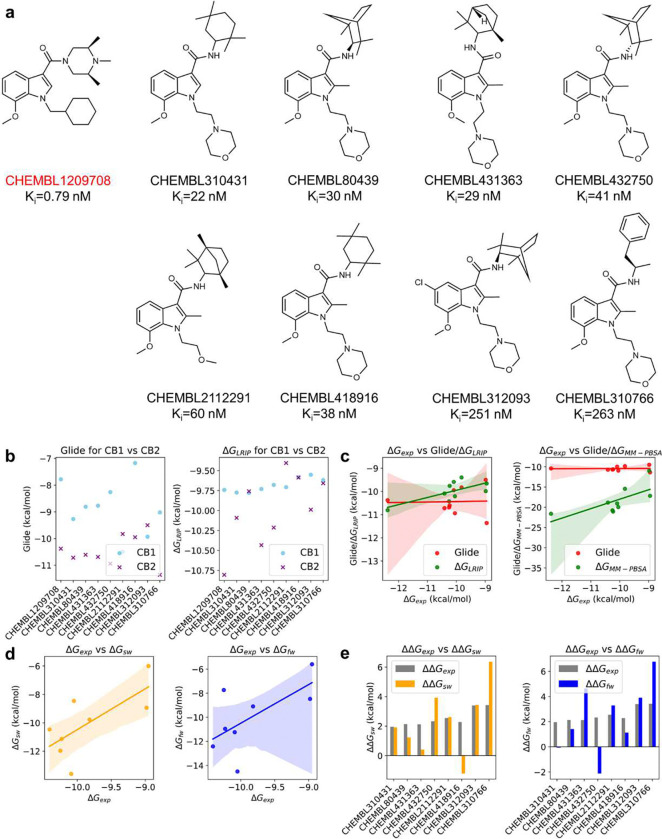
Validation of the applied computational methods in the FSGLD pipeline. **a.** Nine ChEMBL compounds containing MCS ii substructure; **b.** Selectivity prediction of Glide docking and LRIP energy prediction for ChEMBL compounds targeting CB1 and CB2; **c.** The Pearson correlation between $\Delta G_{\text{exp}}$ versus Glide docking score (red dots), $\Delta G_{\text{exp}}$ versus $\Delta G_{\text{LRIP}}$ (green dots) and $\Delta G_{\text{exp}}$ versus $\Delta G_{MM-PBSA}$ (green dots); **d.** The Pearson correlation between $\Delta G_{\text{exp}}$ versus $\Delta G_{sw}$ (yellow dots) and $\Delta G_{\text{exp}}$ versus $\Delta G_{fw}$ (blue dots); **e.** The comparison of two TI protocols in relative binding free energy calculation.

In this section, we applied a set of computational tools in FSGLD and bioassays to assess the quality of DRUG-GAN generated compounds in Scenario III. These compounds underwent comprehensive validation procedures using a set of hierarchical filters, including molecular docking, LRIP energy prediction, endpoint MM-PBSA free energy calculation, alchemical TI free energy calculation, and experimental radioligand binding assays (detailed in the Methods section). Note that LRIP^40^ is a CB2 target-specific scoring function, which was trained using the ligand-residue interaction profile to achieve efficient yet accurate binding free energy calculations. Only the hits survived from the previous computational filters enter the next one. Nine ChEMBL compounds (Fig. 9a) with measured binding affinity were used as a validation set to evaluate the effectiveness of the FSGLD framework. Nine ZINC compounds containing MCS ii substructure as shown in (Fig. 7b) were evaluated using all the computational filters and binding assay.

As illustrated in **Table S7** and Fig. 9b, the results from both docking and LRIP energy prediction methods indicated that the ChEMBL compounds exhibited a stronger binding affinity for CB2 compared to CB1. This result is expected given those compounds are CB2 ligands. Fig. 9c–9d highlighted the strong correlations between experimental versus predicted binding energies across several advanced computational approaches, including LRIP, MM-PBSA and TI. All of them outperformed the Glide docking method which has a Pearson correlation coefficient (R) of 0.03. On the contrary, the R values are both 0.69 for LRIP and MM-PBSA methods. As to the most rigorous method, TI, achieved R values of 0.71 if using the single-window (sw) schedule, and 0.64 if using the full-window (fw) schedule. Based on these results, we concluded that LRIP is an accurate and efficient method to enrich top hits from docking screening, and performance our newly proposed sw-TI method achieved a better performance than regular TI.

In the following, we provided more detailed comparisons between the two TI protocols with CHEMBL1209708 as the reference state molecule (Fig. 9e). For sw-TI, only CHEMBL418916 displayed a negative $\Delta \Delta G_{sw}$ value, contrary to the experimentally observed positive $\Delta \Delta G_{\text{exp}}$. In contrast, fw-TI produced incorrect predictions for two compounds, CHEMBL310431 and CHEMBL2112291, where the predicted $\Delta \Delta G_{fw}$ values were opposite to the experimental $\Delta \Delta G_{\text{exp}}$ values. Thus, sw-TI achieved more accuracy in predicting the direction of calculated free energy changes than fw-TI. The better performance of sw-TI measured by both prediction accuracy and correlation to experimental data, in addition to the significantly reduced computational cost (the cost of sw-TI is only 1/*n* of fw-TI, where *n* is the number of windows in fw-TI), we concluded that in certain cases, sw-TI can serve as an alternative filter of regular TI in the drug screening pipeline.

**Fig. 10:**
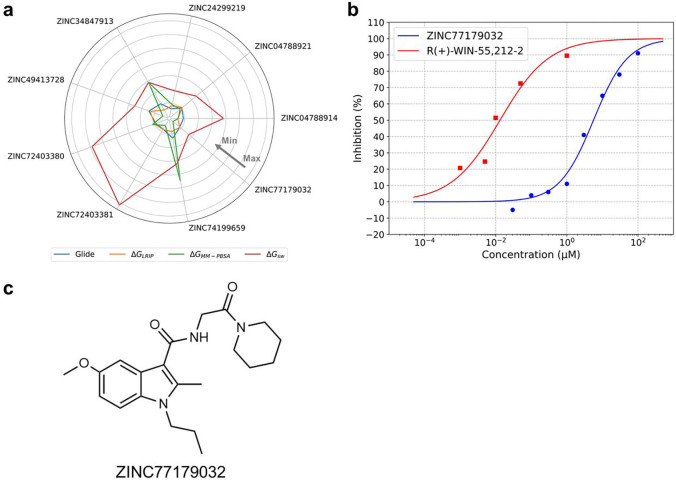
Validation of FSGLD pipeline using ZINC compounds. **a**. Radar plot of the calculated binding energies of ZINC compounds utilizing Glide docking, LRIP energy prediction, MM-PBSA and sw-TI methods; **b.** Concentration-response curves of ZIN77179032 and control compound R(+)-WIN-55,212–2 in radioligand binding assays of CB2 receptor; **c.** the chemical structure of ZINC77179032.

After we validated the computational methods within the FSGLD pipeline using a set of ChEMBL compounds, we applied the complete workflow to ZINC compounds containing MCS ii. After applying all the computational filters, i.e. Glide docking, LRIP energy calculation, MM-PBSA and sw-TI sequentially, nine compounds shown in Fig. 10a were acquired for binding assay with an aim to further validate the FSGLD pipeline. The calculation results of the four computational filters were summarized in **Table S8**. Through this rigorous screening process, ZINC77179032 emerged as the most promising candidate, exhibiting the strongest predicted binding affinities with both MM-PBSA and TI methods.

Among the nine compounds, ZINC77179032 demonstrated 65% inhibition of 10 μM R(+)-WIN-55,212–2, the control compound in radioligand binding assay. Further dose-response assay revealed that ZINC77179032 can bind to CB2 receptor with K_i_ of 3.77 μM and an IC_50_ of 5.62 μM (Fig. 10b). To further evaluate the potential of ZINC77179032 as a selective lead compound, we also conducted radioligand binding assay targeting CB1. There was no inhibition observed for ZINC77179032 binding to CB1 at a concentration ranged from 0.03 to 100 μM, confirming its high CB2 selectivity. These findings affirm the efficacy of the FSGLD pipeline in identifying novel, potent and target-specific compounds to enter the next stage of drug discovery and development.

## 3. Discussion

In this work, we presented Full-Spectrum Generative Lead Discovery (FSGLD), a novel and comprehensive pipeline to efficiently and accurately identify potential lead compounds by incorporating our developed generative models. FSGLD represents a comprehensive, end-to-end approach that leverages generative modeling to expand chemical space as well as a series of validation approaches to efficiently and accurately identify high-potential lead compounds. The computational validation workflow consists of a set of hierarchical filters including molecular docking, MD simulations, LRIP energy prediction, MM-PBSA free energy calculations, and TI. FSGLD pipeline provides a highly scalable and efficient framework, significantly reducing both the time and cost of identifying viable new drug candidates. To the best of our knowledge, this is the first pipeline to advance rational drug discovery by integrating generative modeling, *in silico* molecular screening and *in vitro* experimental validation.

The central component of the FSGLD pipeline is the generative model, DRUG-GAN, which applies dcGAN and cdcGAN to *de novo* design drug-like and target-biased molecules. The DRUG-GAN model was developed and implemented across three distinct scenarios: I. generation of random drug-like compounds, II. generation of target-biased compounds, and III. generation of target-biased compound series containing MCS features. In this work, we have demonstrated that DRUG-GAN models, which use MACCS as molecular descriptor, offer an efficient and effective alternative to more popular approaches including SMILES strings and 3D structure generation. Although MACCS cannot be directly converted into 3D structures, we can apply similarity search on the designed FPs to enhance the efficiency of molecular generation and address the synthetic feasibility issue.

For all three scenarios, DCNNs were applied for both the discriminator and generator of the GAN models. Our investigation into the impact of various discriminator architectures on the discriminator performance revealed that AlexNet-based architecture achieved the best performance in distinguishing active from inactive molecular FPs. Notably, models based on VGGNet11, VGGNet13, and VGGNet16 architectures exhibited poorer performance, indicating that the number of convolutional layers in DCNN models does not necessarily correlate with improved accuracy. This finding highlights the importance of architecture-specific tuning in building effective machine learning models for drug discovery. It is undeniable that the construction of a DCNN model is case-specific and is susceptible to the setting of convolutional layers and hyperparameters. The good performance of the discriminator makes it capable of accurately distinguishing reals and fakes.

Following the construction of the generator model with the same architecture, DRUG-GAN was tested for the generation of random drug-like compounds (Scenario I). Our comparative analysis showed that DRUG-GAN outperformed established models such as ORGAN, OR(W)GAN, and ORGANIC across several key performance metrics, particularly when we employed the 70% similarity threshold in database search. Compounds generated by DRUG-GAN exhibited superior drug-like properties, with properly ranged MW, balanced lipophilicity, and optimal QED scores. The 70% similarity threshold provided the best balance between molecular diversity and drug-likeness, as reflected by favorable distributions of MW, TPSA and QED scores. This balance, along with enhanced structural diversity, makes DRUG-GAN an optimal tool for *de novo* drug-like compound generation. For scenario II, DRUG-GAN demonstrated its efficacy in generating target-biased compounds, specifically for CB2. The model successfully retrieved active CB2 compounds from the ChEMBL database, as well as novel candidates from the ZINC database. These subsequent validation steps confirmed the model’s capacity to identify viable leads with high selectivity for the target-specific receptor, offering significant potential for further development. Scenario III extended the application of DRUG-GAN to generate target-biased compound series which contain common MCS features. The resulting compounds from the database searches were subjected to a set of hierarchical computational filters and finally in vitro binding assay.

To prioritize the DRUG-GAN designed compounds, FSGLD pipeline applies a comprehensive screening process to prioritize designed compounds to enter the next stages of validation with more advanced modeling method and binding assay. By this way, we can identify the most promising candidates reliably and efficiently. We have demonstrated the superiority of the free energy-based filters, i.e., LRIP, MM-PBSA and TI, over traditional molecular docking. Notedly, LRIP achieves a comparative performance of MM-PBSA with a cost comparable to that of molecular docking, thus, can bridge the gap between the efficient docking method and the rigorous free energy methods (such as MM-PBSA and TI) which require conformational samplings. Interestingly, we have found that the regular TI protocol can be simplified without necessarily harming the prediction results. In the case of CB2, single-window TI protocol surprisingly outperformed the more complex 9-window Gaussian quadrature-based protocol. Thus, under certain conditions, the simplified TI protocol should be considered as a part of computational workflow in drug discovery.

In conclusion, the application of our FSGLD pipeline represents a successful and complete integration of AI-driven compound generation with comprehensive *in silico* and *in vitro* validation procedures, providing an efficient and accurate platform for lead discovery. By combining generative models with rigorous molecular evaluation techniques, FSGLD represents a major advancement in the ability to identify and validate potential lead candidates, offering significant time and cost savings for both academic researchers and industry professionals.

## 4. Methods

### 4.1 Dataset Preparation

For Scenario I, the active dataset was compiled by collecting 7002 drugs from DrugBank (https://go.drugbank.com/) based on the Lipinski’s rule of five^52^ and diversity selection. For the decoy set, we collected 100,000 druglike screening compounds with molecular weight (MW) and logP that meet with the criteria of Lipinski’s rule from ZINC database (https://zinc.docking.org/). To balance the sample size in active and inactive (decoy) sets, we only selected a subset that matches the general distributions of the two molecular properties, MW and logP, for the 100,000 decoys. To do this, we performed principal component analysis (PCA)^53^ to reduce the two-dimension features (MW and logP) into a single dimension. Then those inactive compounds were sampled based on the density of new distribution of the reduced-dimension feature. Finally, 9998 molecules were selected to form the decoy set. To train and validate the discriminator of dcGAN, all selected active (7002) and inactive molecules (9998) were partitioned into training set and test set using 10-fold cross validation method^54^.

For the target-biased compound generation in Scenarios II and III, we collected 1896 active (K_i_/IC50 < 100 nM) and 2996 inactive (K_i_/IC50 ≥ 100 nM) CB2 compounds from ChEMBL database to train the cdcGAN model. In addition, we also manually checked the chemical structures of collected CB2 active compounds and identified three most common maximal common substructures (MCSs) as shown in Table 4 and **Fig. S1**. During the training, all molecules are represented by MACCS FP (168 bits) by utilizing Open Babel program version 2.3.1 (http://openbabel.org)^55^. For further validation, X-ray structure of CB1 (entry code: 5XRA) and constructed homology model of CB2 were downed and prepared as described elsewhere ^56^.

### 4.2 Model Architecture

**Fig. 11:**
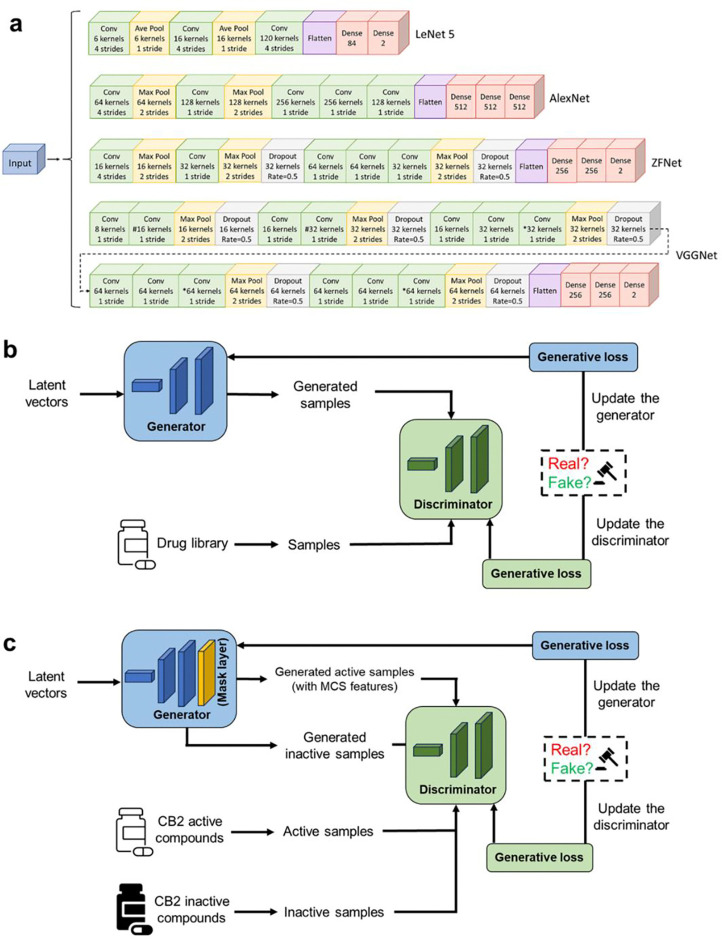
The schematic view of DRUG-GAN architectures. **a.** The schematic depiction of DCNN architecture. **b.** GAN architecture for *de novo* design of drug-like molecules. **c.** GAN architetures for *de novo* design of target-specific (CB2) with/without preserved maximum common structure features.

DCNN is a deep neural network that is mainly designed for processing image and speech data. In DCNN, to construct the model, convolutional layers were implemented sequentially and each neuron in a convolutional layer receives input from the output of the previous layer. The model was trained to adjust hyperparameters including padding, strides, filter size, learning rate, etc. For the discriminator of a DCGAN model, we used a dense network architecture for the input layer, followed by different convolutional layers and dense layers to construct the DCNN model. In detail, four major types of DCNN architectures were explored in our work, which are LeNet-5^57^, AlexNet, ZFNet^32^ and VGGNet^33^. To be noted, VGGNet-based architectures have three types with different number of weight layers. VGGNet11-based and VGGNet13-based models are modified versions of VGGNet16 by dropping off the pound sign and the asterisk sign levels for VGGNet 11 and VGGNet13, respectively. The details of those architectures are described in Fig. 11a. Before passing to the dense layers, input features were flattened. Hyperparameters, such as pool size, kernel size, padding (*causal, same, valid*) and activation (*tanh, softmax, relu*) were tuned and optimized during the training process. As for the model compilation of the discriminator, the loss function was set to *categorical crossentropy*, kernel regularizer to *L2 penalty*, and the optimizer to *Adam*.

Then the generator was created by reversing the convolution process of the discriminator. GANs are generative models that allow for learning data distribution without the need to explicitly define a likelihood function. GANs consist of two competing neural networks: a generative model (G) that maps random variables (z) drawn from a prior distribution to the data distribution, and a discriminative model (D) that distinguishes between real data $\left(P_{\mathrm{data}}\right)$ and data generated by G. The two components of a GAN model, the generator and discriminator, were trained simultaneously to achieve an objective described by the following formula.

$$\underset{G}{min}\;\underset{D}{max}\;E_{x\sim P_{\mathrm{data}\;}(x)}[log\;D(x)]+E_{z\sim P_{z}(z)}[log(1-D(G(z)))]$$

The two networks engage in a minimax game, where G aims to produce realistic samples to fool D, while D learns to correctly classify real data versus generated ones. This adversarial process allows G to improve its generation of realistic data over time.

Fig. 11b–c is a schematic diagram for both dcGAN and cdcGAN models with different objectives of molecular generation. Overall, a discriminator is trained with real molecules utilizing the DCNN model to identify if a molecule fingerprint comes from the generator. The generator consists of four convolutional layers utilizing batch normalization and *leaky ReLU* activation function. For the compilation of both generator and DCGAN model, the loss function was set to *categorical crossentropy* and the optimizer was again set to *Adam,* with the *learning rates* being set to 10^−6^ for the discriminator and 10^−3^ for the generator.

In Scenario III, the objective is to design novel CB2 molecules containing MCS structures. To achieve this goal, we introduced a mask layer, *lambda layer*, into the architecture of the generator network. This mask layer is designed to ensure that the generated CB2 FPs retain the feature bits of the MCS, so that those MCS structures will appear in the designed molecules. To construct this mask, we first delineated the MCS features from a predefined dataset (MCS i, MCS ii, MCS iii), creating a binary variable (mask) where MCS feature bits were set to 1, and an inverse variable (unmask) where non-MCS feature bits were set to 1. This approach can be mathematically represented as:

$$mask[i]=\left\{\begin{array}{l} 1\;\mathrm{if}\;i\in \mathrm{MCS}\;\mathrm{features}\\ 0\;\mathrm{if}\;i\notin \mathrm{MCS}\;\mathrm{features} \end{array}\right.\;\mathrm{unmask}[i]=1-\mathrm{mask}\;[i]$$

In the architecture of the generator, the lambdalayer was appended to the last convolutional layer, and the output of the lambdalayer is calculated using the following formula:

lambdalayer(x)=x×unmask+mask

Where x is the raw output of the last convolutional layer. This operation ensures that the MCS feature bits are preserved in the generated FP, while allowing flexibility in other bits. The output from this lambdalayer thus becomes the final output of the generator, ensuring that each generated fingerprint adheres to the MCS constraints. The incorporation of this mask layer represents a significant enhancement to the basic cdcGAN architecture, allowing for the generation of chemically relevant compounds with desired structural features, essential in drug lead optimization processes.

### 4.3 Training and Sampling

First, the discriminator was trained independently using authentic molecular FPs and decoy FPs. All given FPs were separated into 10 folds for training and testing using the StratifiedKFold, a Scikit-learn^58^ module built in Python.^59^ Each of the ten subsets was iteratively taken as the test set to evaluate the model generated using the rest of the nine subsets. All the DCNN architectures shown in Fig. 11a were evaluated with an aim to identify the best-performed one for composing the DRUG-GAN model architecture. For each training, the number of training epochs was set to 100.

Next, the dcGAN model was trained with the objective of generating drug-like FPs (Scenario I). For each epoch of the dcGAN model training process, there are two training steps. In the first step, only the discriminator was set to be trainable. We trained the discriminator to minimize the discriminative loss. Meanwhile, the generator began to generate molecular FPs with the noise input. Drug molecular FP (positive label) and the generated FP (negative label) were given to the discriminator to make the classification. For the second step, the trained discriminator in the first step was frozen and the generator was trained to minimize generative loss. The generator still starts with the noise input to generate molecular FPs. The real and the generated data were given to the discriminator for the classification together with positive labels. The GAN model was trained for 800 epochs to ensure both the discriminator and generator converged. The batch size was set to 32 for the mini-batch training. Model construction and training for dcGAN was conducted using Keras module^60^ and Tensorflow^61^ as the backend built in Python.

For the cdcGAN model training (Scenarios II and III), the same training procedure and parameters for dcGAN models were adopted directly, to standardize the training frameworks of DRUG-GAN models in all scenarios. The cdcGAN’s unique aspect was its incorporation of inactive CB2 compounds as conditional labels, a feature that significantly diverged from the dcGAN modeling. This conditioning was critical in guiding the model’s focus towards the synthesis of active CB2 compounds. Model construction and training for cdcGAN models were conducted using Keras module^60^ and Tensorflow^61^ as the backend built in Python.

### 4.4 Evaluation methods for generated molecules

#### Model Performance.

The performance of the DCNN model for the discriminator was evaluated by using a series of metrics, which are AUC of receiver operating characteristic (ROC) curve, accuracy, F1-score, precision, recall and confusion matrix. The AUC, accuracy, F1-score, precision and recall all range from 0 to 1, with 0 indicating the worst and 1 the best performance. Specifically, for ROC, a random model has an AUC of 0.5 theoretically, and a good classifier has AUC of 0.8 or higher. For the confusion matrix of testing data, the sum of true positives (TP), true negatives (TN), false positives (FP) and false negatives (FN) is 1400, one-tenth of the total amount of real drugs (7002) and compounds decoys (9998). Since the dataset was split into 10 folds using the cross-validation method, the average values of all metrics for different DCNN architectures were calculated and compared.

#### Drug-like compounds.

To assess the performance of dcGAN model, we performed a quantitative analysis from two aspects for the 10,000 molecular FPs generated from the generator after the training process. To directly evaluate the generated MACCS, we introduced four metrics for evaluation: (1) Uniqueness: the ratio of the number of unrepeated molecules to the number of valid molecules; (2) Novelty: the ratio of the number of molecules which are not included in the training set to the number of unique molecules; (3) Diversity: $\left(1.0-\frac{1}{N}\sum_{i,j>i}TS_{ij}\right)$ for all N molecule pairs (i,j>i) in the test set, where $TS_{ij}$ is measured by the Tanimoto score (TS)^62^ of molecules i and j. (4) Similarity: The average of $TS_{ij}$ of all molecule pairs, with molecule i being a generated molecule, and molecule j in the training set.

Additionally, the performance of our dcGAN model was compared with three published models, ORGAN, ORGANIC and OR(W)GAN for which the Wasserstein-1 W distance was implemented for the discriminator ^63^. We utilized 10 and 250 epochs to train the discriminator and the generation, and 100 epochs to train the GAN model. After training, 10,000 SMILES were sampled from three published models, respectively. The four metrics were also computed for the MACCS converted from those 10,000 SMILES.

For our DRUG-GAN model, we also need to evaluate the structural similarity between the generated FPs and the retrieved compounds from molecular databases. We first collected compounds that were no less than 70%, 75% and 80% structurally similar to the dcGAN-generated FPs by searching the ZINC database. Next, the model performance of dcGAN was further evaluated by comparing drug-likeness of the collected compounds against the valid SMILES generated by three published models. For drug-likeness evaluation, we focused on MW, logP, topological polar surface area (TPSA) and Quantitative Estimate of Drug-likeliness (QED) properties^64^.

#### Target-biased compounds.

As for the evaluation of cdcGAN model performance, 10,000 molecular FP were generated and sampled. Compounds from the ChEMBL and ZINC databases with similarity scores of at least 70%, 75%, 80%, and 85% compared to the sampled CB2 active FPs were then identified. The top-selected ZINC candidates underwent molecular docking against both the CB1 and CB2 receptors for further validation.

#### Compound series.

For each MCS substructure, 10,000 special molecular FPs were generated and sampled. Similarly, compounds from the ChEMBL and ZINC databases with similarity scores of at least 70%, 75%, 80%, and 85% compared to the sampled CB2 active FPs were then identified. Particularly, for those identified compounds containing a MCS substructure, we converted them into 1024-bit FP2 FPs to perform the second-round similarity search from the same database, making sure the searched compounds truly contain the MCS substructure. Then molecular docking for the top-selected ZINC candidates with different MCS features targeting to both the CB1 and CB2 receptors were performed.

Next, we evaluated those hits using a set of hierarchical filters in FSGLD. The computational details on those filters, including molecular docking, LRIP energy prediction, MM-PBSA and TI method, as well as the experimental radioligand binding assays, were presented in the supplementary text.

## Figures and Tables

**Table 1.** Evaluation of DRUG-GAN and Published Generative Models on Generating Valid Samples.

Model	Uniqueness	Novelty	Diversity	Similarity
ORGAN	0.66	0.83	0.77	0.20
OR(W)GAN	0.39	0.84	0.63	0.19
ORGANIC	0.76	0.82	0.76	0.22
DRUG-GAN	1.00	1.00	0.73	0.25

For each model, the performance metrics were averaged over 10,000 samples.

**Table 2:** Evaluation of DRUG-GAN and Published Generative Models on Generating Druglike Molecules.

Model	MW	logP	TPSA	QED
DRUG-GAN (TS ≥70%)	382.92(63.70)	1.984(1.68)	76.01(22.14)	0.66(0.16)
DRUG-GAN (TS ≥75%)	392.98(65.62)	1.59(1.80)	88.32(25.53)	0.61(0.16)
DRUG-GAN (TS ≥80%)	396.15(66.87)	0.37(2.22)	113.88(26.95)	0.50(0.14)
ORGAN	179.24(66.57)	2.26(1.58)	33.57(24.92)	0.60(0.13)
OR(W)GAN	267.85(124.18)	3.26(1.94)	38.35(32.86)	0.62(0.17)
ORGANIC	177.33(66.38)	1.30(1.94)	55.68(34.22)	0.52(0.14)

For each model, the molecular properties were averaged over 10,000 samples. The value in a parenthesis refers to the standard deviation of the molecular property.

**Table 3.** The number of searched compounds using different similarity criteria from ChEMBL and ZINC databases.

Database	TS≥0.70	TS≥0.75	TS≥0.80	TS≥0.85
ChEMBL	212093	38771	2517	186
ChEMBL (100 ≤ MW ≤ 500)	151211	28669	2053	169
ZINC	1583230	270875	13652	161

**Table 4.** Summary of substructure and similarity search using MACCS fingerprints in Scenario III.

MCS Feature	Database and Subset	TS≥0.70	TS≥0.75	TS≥0.80	TS≥0.85
MCS i 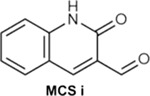	ChEMBL	4348	1012	17	0
	ChEMBL (MW: 100–500)	2240	320	13	0
	ZINC	15219	2460	75	0
MCS ii 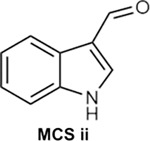	ChEMBL	14355	2626	177	2
	ChEMBL (MW: 100–500)	6382	1093	77	2
	ZINC	89105	15912	911	13
MCS iii 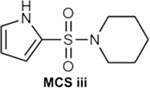	ChEMBL	602	284	58	18
	ChEMBL (MW: 100–500)	323	165	29	0
	ZINC	3917	1330	170	5

## Data Availability

The data that support the findings of this study are available within the main text and the Supplementary Information.
